# Supplementary material for: Critical non-Hermitian skin effect
Source: Nat Commun. 2020 Oct 30;11:5491. doi: 10.1038/s41467-020-18917-4 (PMC7603343; doi:10.1038/s41467-020-18917-4)
Supplement: Supplementary file 1 — Supplementary Information [file 41467_2020_18917_MOESM1_ESM.pdf]

Supplementary Information - Critical non-Hermitian Skin Effect

Linhu Li,* Ching Hua Lee,† Sen Mu,‡ and Jiangbin Gong§

* rubilacxelee@gmail.com
† phylch@nus.edu.sg
‡ senmu@u.nus.edu
§ phygj@nus.edu.sg

SUPPLEMENTARY NOTE 1: RECIPROCAL REALIZATION OF THE TWO-CHAIN MODEL

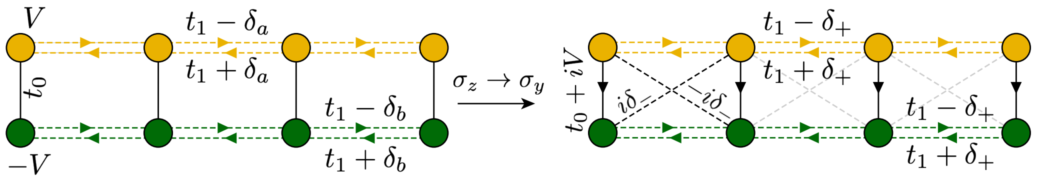

Supplemental Figure 1. The two-chain model with a rotation of the basis. The rotated lattice has only reciprocal hoppings when $\delta_+ = V = 0$.

Here, we discuss how the CNHSE, which requires subsystems having different NHSE decay lengths, can in fact be realized with reciprocal models that are more easily realizable in experiment. In the two-chain model, the Hamiltonian can be rewritten in the form of Pauli matrices as

$$\begin{aligned} h(z) = &[t(z + 1/z) + \delta_+ \sin k(z - 1/z)]\sigma_0 \\ &+ t_0\sigma_x + [V + \delta_-(z - 1/z)]\sigma_z, \end{aligned} \tag{1}$$

with $\delta_\pm = (\delta_a \pm \delta_b)/2$. Here δ_+ describes the equivalent part of non-Hermiticity acting on the two chains, which shall induce the same NHSE to them. The critical behavior and transition of NHSE occurs only with nonzero δ_-, which induces band-dependent NHSE along the two chains. As shown in Fig. 1, $\delta_\pm$ can be divided into different couplings with a rotation of pseudospin $\sigma_z \to \sigma_y$, and the rotated Bloch Hamiltonian $h_r(k)$ satisfies $h_r^T(k) = h_r(-k)$ at $\delta_+ = V = 0$. Under this condition, the rotated system is reciprocal, and thus provides convenience for experimental realization such as RLC circuit lattices. Such a rotation is represented by a unitary transformation applied to the Hamiltonian, which only redefines the two sublattices within each unit cell, but does not change the eigenenergies and the spatial profile of eigenstates of the system. Note that in this particular regime, the two decoupled chains have degenerate eigenenergies, and the associated NHSE has very weak skin localization (i.e. $\kappa \approx 0$) once the two chains are coupled by t_0. This peculiar feature is analogous to the NHSE suppression by an additional coupling discussed in Ref. [2].

SUPPLEMENTARY NOTE 2: NON-MONOTONICITY OF CONVERGENCE TOWARDS E_∞

In Fig. 2, we illustrate the PBC-OBC spectral flow [1] of the two-chain model with different parameters, by rescaling the amplitudes of the hopping across the boundary as $t_1 \pm \delta_{a,b} \to c(t_1 \pm \delta_{a,b})$, and tuning c from 1 (PBC) to 0 (OBC). We can see that in the decoupled limit, each of the two PBC bands (red or blue) merges with itself along the real axis when approaching OBC limit [Fig. 2(a,d,e)]. On the other hand, in the coupled regime of Fig. 2(b,c,f), each band first flows toward the real axis, but then "turns back" and merges with the other band, forming a central-loop structure. In this process, the PBC bands do not necessarily go monotonically closer to OBC spectrum that reflects the GBZ solutions. A systematic study of the interplay between the switching off of boundary couplings (PBC-OBC interpolation) and subsystem coupling (t_0) is deferred to future work.

SUPPLEMENTARY NOTE 3: ANOMALOUS SCALING OF ENTANGLEMENT ENTROPY

The scaling behavior of Fermionic entanglement entropy (EE) S depends qualitatively on the nature of the phase, increasing as $\frac{1}{3}\log N$ at an ordinary critical point, decreasing possibly as a negative multiple of $\log N$ at a critical exceptional point [3], and saturating at a gapped or decoupled scenario. Since N itself can drive phase transitions in our case of the CNHSE, we expect the scaling of S to interpolate and make transitions through distinct behaviors.

For free Fermions in a many-body state $|\Psi\rangle$, the (biorthogonal) EE [4, 5] for a chosen entanglement cut can be computed via

$$S = -\sum_j [c_j \log c_j + (1 - c_j)\log(1 - c_j)], \tag{2}$$

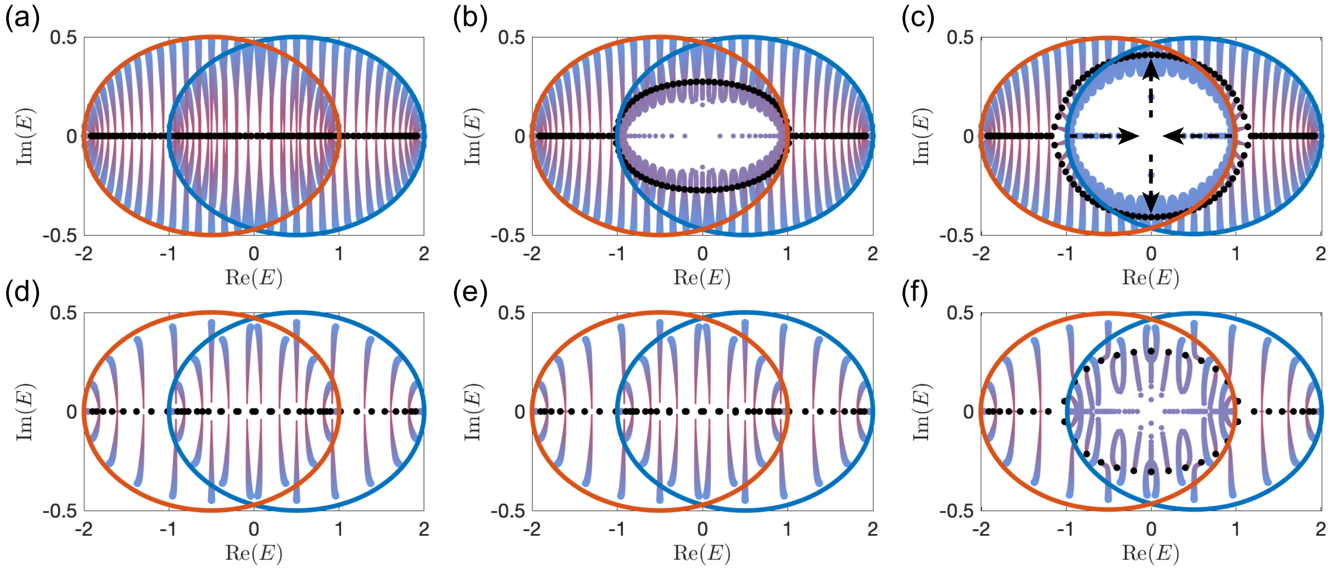

Supplemental Figure 2. Spectral flow of the two-chain model. (a-c) for $N = 60$ unit cells, (d-f) for $N = 20$ unit cells. From left to right, the inter-chain coupling is $t_0 = 0, 2 \times 10^{-4}, 0.1$ respectively. Other parameters are $t_1 = 0.75$, $\delta_a = -\delta_b = 0.25$, and $V = 0.5$. Red and blue circles are periodic-boundary spectra obtained from the Bloch Hamiltonian, black dots are open-boundary spectra, and blue-purple curves are the spectral flow from a periodic-boundary system to an open-boundary one. In the coupled regime of (b,c,f), two points of periodic-boundary bands on the real axis first flow toward zero energy, then rapidly separate along the imaginary axis, as shown by the arrows in Fig. 2(c).

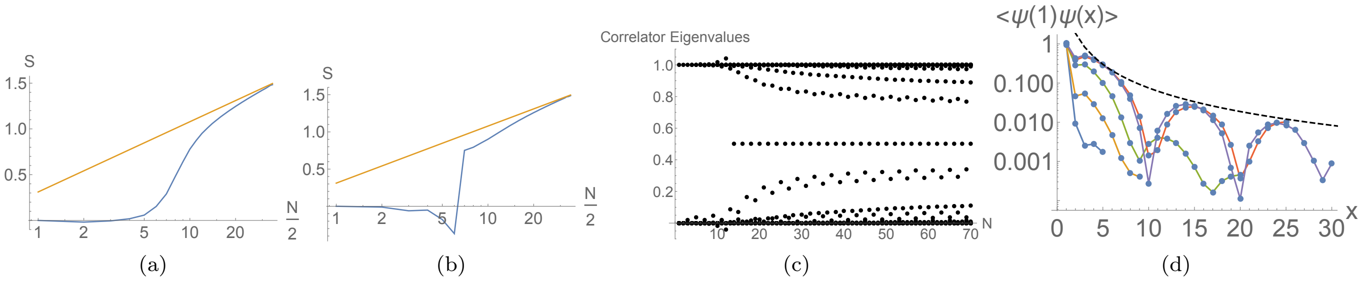

Supplemental Figure 3. (a,b) Scaling of S with odd/even N (blue) for a half-filled OBC $H_{2\text{-chain}}$ with real-space cut at $\lfloor \frac{N}{2} \rfloor$ and parameters $t_1 = 0.58$, $V = 1$, $t_0 = 0.4$ and $\delta_a = -\delta_b = 0.25$ (same as Fig. 3a of the main text). At small N, S is almost vanishing for odd system sizes, and negative for even ones. At larger N, both odd and even cases display a tendency towards the expected $S \sim \frac{1}{3}\log N$ critical behavior (yellow). (c) The corresponding correlator eigenvalues c_j, showing how the system makes transitions to critical behavior with a single $c_j = 1/2$ (and other eigenvalues slowly approaching it) only beyond $N \approx 10$. Before that, the system is essentially decoupled. (d) The corresponding two-Fermion correlation at $N = 10, 18, 40, 50, 60$ (blue,brown,green,puple,red), with rapid exponential decay for small N and power-law decay for large N (Black dashed curve shows N^{-1} decay for reference).

where the c_j's are the eigenvalues of the 2-particle correlator $C = PQP$ [6–8]. Here P is the projector implementing the entanglement cut and $Q = \sum_{\mu \in occ.} |\psi_\mu\rangle\langle\psi_\mu|$ is the single-body biorthogonal projector onto the set of basis states $|\psi_\mu\rangle$ occupied by the many-body state $|\Psi\rangle$. In a perfectly unentangled case, $c_j = 0$ or 1 only, giving rise to a vanishing EE. With increased entanglement, c_j becomes closer to $1/2$, attaining the latter when the sector j is fully entangled. In the biorthogonal setting, it is possible for c_j to take values outside of $[0, 1]$ since $|\psi_\mu\rangle$ is not the complex conjugate of $\langle\psi_\mu|$, leading to negative or even imaginary contributions to S [3].

In Fig. 3, we observe a crossover from a decoupled regime to a critical regime when N increases. S also exhibits non-universal negative values for certain even N [Fig. 3(b)], a behavior resulting from $c_j \notin [0, 1]$ for a few of these N. In real space, the two-Fermion correlator C decays rapidly for small N, but interestingly decays more slowly like x^{-1} for larger N when the system becomes gapless. As such, correlators generally become enhanced in larger systems where the effects of coupling become amplified by the CNHSE.

SUPPLEMENTARY NOTE 4: MAPPING BETWEEN THE SSH MODEL AND TWO NON-RECIPROCAL 1D CHAINS

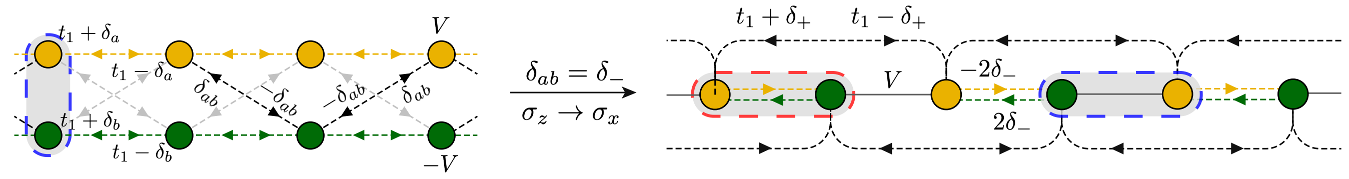

Supplemental Figure 4. Transforming the two-chain model with non-reciprocal cross couplings to a SSH model with non-reciprocal inter-cell couplings and second-nearest-neighbor couplings. The different parameters in the two panels are connected through $\delta_\pm = (\delta_a \pm \delta_b)/2$. Blue dash lines indicate a unit cell before and after the rotation, and red dash line indicates an alternative choice of unit cell with a shift of one lattice site, with which the non-reciprocal couplings of $2\delta_-$ can be further transformed into on-site gain and loss.

In the main text we have considered a two-chain model with both intra-chain and inter-chain couplings being non-reciprocal, described by the Hamiltonian

$$H_{\text{CNHSE-SSH}}(z) = [i\delta_{ab}(z+1/z)]\sigma_y + [V+\delta_-(z-1/z)]\sigma_z + [t_1(z+1/z)+\delta_+(z-1/z)]\mathbb{I} \quad (3)$$

with $\delta_\pm = (\delta_a \pm \delta_b)/2$. In the parameter regime with $\delta_{ab} = \delta_-$, through a rotation of basis $\sigma_z \to \sigma_x$, this Hamiltonian becomes

$$H_r(z) = \begin{pmatrix} t_1(z+1/z)+\delta_+(z-1/z) & V+2\delta_- z \\ V-2\delta_-/z & t_1(z+1/z)+\delta_+(z-1/z) \end{pmatrix}. \quad (4)$$

This Hamiltonian describes a SSH model with non-reciprocal inter-cell couplings and second-nearest-neighbor couplings, as illustrated in Fig. 4. In the main text we have chosen $\delta_a = -\delta_b$, so that $\delta_+ = 0$ and the second-nearest-neighbor couplings are Hermitian. In this parameter regime, by reorganizing the unit cell as the red dashed line in Fig. 4 (shifting one lattice site), we can see that the rotated model is equivalent to the non-reciprocal SSH model studied in Refs. [9, 10] etc. with a uniform second-nearest-neighbor couplings, described by the Hamiltonian

$$H_r'(z) = \begin{pmatrix} t_1(z+1/z) & V/z+2\delta_- \\ Vz-2\delta_- & t_1(z+1/z) \end{pmatrix}. \quad (5)$$

Finally, by applying another rotation of basis $\sigma_y \to \sigma_z$, the system can be further transformed into a ladder model with non-Hermiticity being only on-site gain and loss [11, 12].

Note that in the main text we have considered the case with $\delta_{ab} \ll \delta_-$. In the SSH model, this inequality corresponds to some extra longer-range couplings. Also note that the reorganization of unit cells (blue to red dash lines in Fig. 4) corresponds to a different lattice structure where the first and last lattice sites are coupled by V instead of $\pm\delta_-$. Under OBCs, these two choices of unit cells will result in different behaviors of topological edge states.

SUPPLEMENTARY NOTE 5: TOPOLOGICAL PROPERTIES OF THE CNHSE SSH MODEL

a. Edge states in a line gap

Here we consider the CNHSE-SSH model discussed in the main text, but with a stronger inter-chain coupling strength $\delta_{ab} = 0.15$. We can see in Fig. 5 that the system has a narrow real line-gap at small $N = 10$, a point-gap at $N = 20$, and an imaginary line-gap at $N = 40$. Degenerate zero-energy edge states emerge in the latter two cases. As the two OBC bands are fully separated from each others in the last case, a Berry phase can be well-defined for each non-Bloch band to characterize the topological properties of this system.

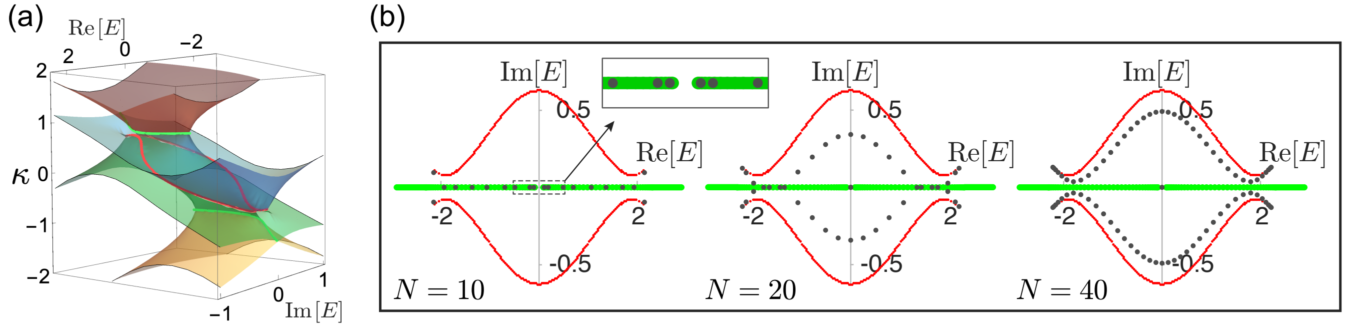

Supplemental Figure 5. (a) κ solutions (red, blue, green, and yellow surfaces) of $f(z, E) = 0$ as a function of the complex energy. Parameters are $\delta_a = -\delta_b = 0.5$, $t_1 = 0.75$, $V = 1.2$, and $\delta_{ab} = 0.15$. Different κ solutions coincide along the green and red dot lines, the latter one giving the OBC skin solutions of the system in the thermodynamic limit. (b) OBC spectra (black dots) at $N = 10$, 20 and 40 unit cells. At small N, the OBC spectrum mostly lies in the real axis and is partially given by the green dot lines in (a), analogous to the skin solutions in the decoupled limit. At larger $|\text{Re}[E]|$, however, the eigenenergies obtained different complex values and form a Y-shape spectrum, matching the OBC skin solutions of the red curves. With increasing system's size, the spectrum continuously approaches these OBC skin solutions.

b. Symmetries

To see the topological properties of our system, we rewrite the Hamiltonian as

$$\begin{aligned} H_{\text{CNHSE-SSH}}(z) &= h_0(z)\mathbb{I} + H_1(z), \\ h_0(z) &= t_1(z + 1/z) + \delta_+(z - 1/z), \\ H_1(z) &= i\delta_{ab}(z + 1/z)\sigma_y + [V + \delta_-(z - 1/z)]\sigma_z. \end{aligned} \tag{6}$$

We can see that $H_1(z)$ possesses the sublattice symmetry (SLS) [13, 14] $\mathcal{S}^{-1}H_1(z)\mathcal{S} = -H_1(z)$ with $\mathcal{S} = \sigma_x$, thus it hosts a Z-type topology and may support zero-energy topological edge states. In Hermitian systems, a term of identity matrix like $h_0\mathbb{I}$ only changes the eigenenergy but not the eigenstates, therefore it does not affect the existence of topological edge states. Essentially, the topological properties are protected by the absence of a third Pauli matrix, giving rise to a quantized winding number of the Hamiltonian. On the other hand, in non-Hermitian systems, OBC topological properties correspond to a PBC non-Bloch Hamiltonian in a GBZ described by the inverse decay length $\kappa(k) = -\log|z|$, which may be affected by $h_0\mathbb{I}$. However, such a modification of $\kappa(k)$ occurs still in the absence of the σ_x term, hence the same topological classification remains, allowing the emergence of topological edge states. For the overall system described by $H(z) = H_{\text{CNHSE-SSH}}(z)$, the absence of σ_x is protected by $\sigma_z H(z)\sigma_z = H^T(z)$, which can be viewed as an effective $\mathcal{PT}$ symmetry constructed by a conjugated time-reversal symmetry $H^T(z) = H(z^*)$ [13] and an inversion symmetry $\sigma_z H(z)\sigma_z = H(z^*)$.

Furthermore, at $\delta_+ = 0$, the total Hamiltonian satisfies

$$\sigma_x H(z)\sigma_x = -H^\dagger(-1/z^*), \tag{7}$$

$$\sigma_z H(z)\sigma_z = H^\dagger(z^*). \tag{8}$$

These two symmetries ensure that the spectrum must be symmetric about imaginary and real axis respectively. More specifically, Eq. (7) [(8)] ensures that for an eigenenergy E_k at quasi-momentum k with inverse decay length $\kappa(k)$, there must be another eigenenergy $E_{k+\pi} = -E_k^*$ [$E_{-k} = E_k^*$] at quasimomentum $k + \pi$ [$-k$] with inverse decay length $\kappa(k + \pi) = -\kappa(k)$ [$\kappa(-k) = \kappa(k)$]. Thus a pair of degenerate edge states associated with the above mentioned symmetries must be fixed at zero energy. A nonzero δ_+ can assign a nonzero energy to the degenerate edge states.

c. Topological invariant for finite-size systems

For non-Hermitian systems, topological invariants are defined for the PBC non-Bloch Hamiltonian in a GBZ, which recovers the OBC spectrum of the system (except for topological edge states). However, in our system with finite size, the OBC spectrum cannot be recovered for the GBZ either of decoupled or coupled limit. Instead, the inverse decay

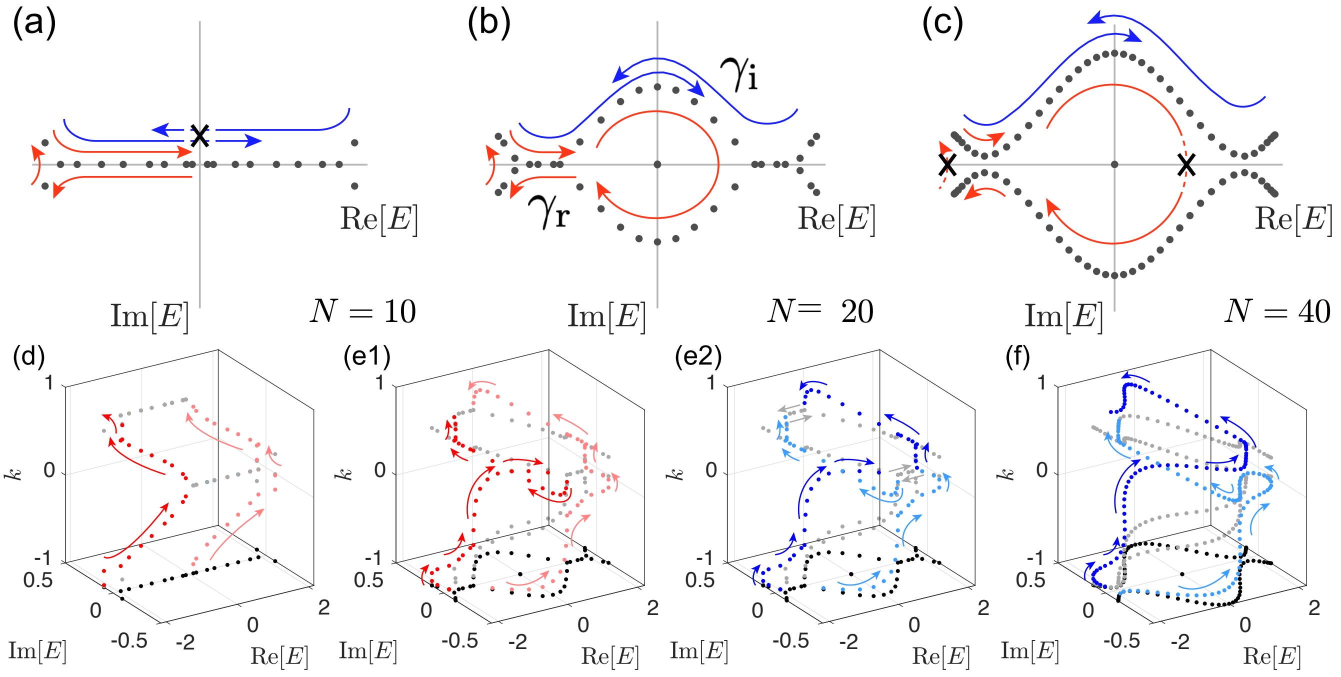

Supplemental Figure 6. OBC spectra as in Fig. 5 with $N = 10, 20, 40$, with red and blue arrows indicating two different routes that connect the eigenenergies as we scan k. In (a) and (c), the two bands are separated by a real line-gap and an imaginary line-gap respectively. Consequently, the Berry phase calculations follow the red arrows in (a) and the blue arrows in (c). On the other hand, the spectrum in (b) has a point-gap, and both routes may be adopted to examine the Berry phase thus defined. (e-g) For completeness, the found k values from Eq. 9 vs OBC eigenenergy is all plotted. Each such OBC eigenenergy E yields four solutions of z, those closest to the GBZ solution of either the coupled or decoupled limit are shown with colored dots, whereas the rest two are shown with gray dots. Black dots are the original OBC spectrum. Red/pink colors distinguish between the two "bands" defined with red arrows in (a-c), and light/dark blue colors distinguish between the two bands defined with blue arrows in (a-c). In (e2), the trajectories of blue arrows "jump" over some gray dots, as shown by the gray arrows. Such a jump is allowed as the spectrum forms a joint central-loop structure, and our Berry phase is defined for discrete quasi-momentum k with separated eigenenergies.

length $\kappa(k)$ for each discrete quasi-momentum k shall be given by some intermediate values between the κ solutions of the two GBZs.

To find such intermediate values of $\kappa(k)$, we first numerically solve the eigenenergies of our system under OBC. We then substitute them into

$$f(z, E) = \det[H(z) - E] = 0 \tag{9}$$

to obtain the quasi-momentum k and the corresponding $\kappa(k)$ for each OBC eigenenergy. Because each OBC eigenenergy E yields four solutions of z, this procedure must be executed with caution. In our investigations, those closest to the GBZ solution of either coupled or decoupled limit are examined to define Berry phase as a topological invariant. With this procedure implemented, we can always operationally obtain the following Berry phase

$$\gamma^{(m)} = -\mathrm{Im} \sum_n \log[\langle \psi_m^L(k_n) | \psi_m^R(k_{n+1}) \rangle], \tag{10}$$

of one band (connected by eigenenergies as we scan discrete values of k from 0 to 2π), with m the band index and n labeling k_n. Indeed, we find $\gamma = \pi$ (0) for the two bands separated by real-axis (imaginary-axis) in Fig. 5(b) at $N = 40$ ($N = 10$), thus verifying the topological nature behind the existence (absence) of in-gap edge states in finite-size systems.

Some additional technical details are of interest because we are attempting to define topological invariants for finite-size systems. The blue and red arrows in Fig. 6 depict our procedure to divide the OBC spectrum into two bands. Specifically, in Fig. 6(a) [(c)], the real [imaginary] line-gap separate the two bands with positive and negative real [imaginary] energies, and the red [blue] arrows indicate one of these two bands can indeed be obtained as we scan discrete values of k from 0 to 2π. Interestingly, in either case, eigenenergies can be also connected by arrows of

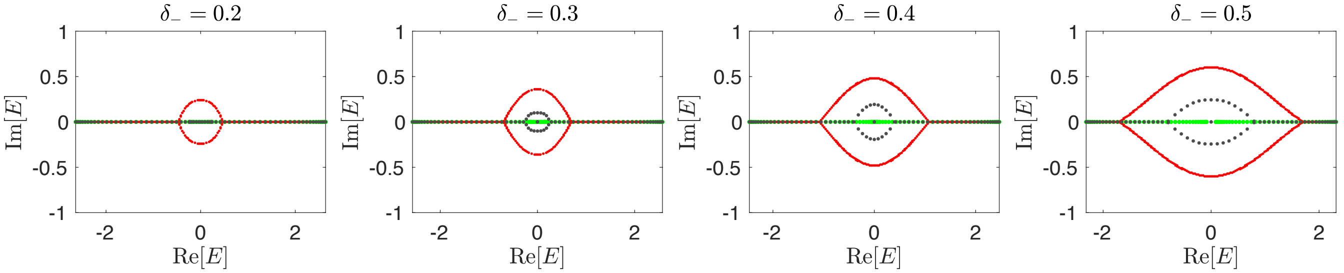

Supplemental Figure 7. OBC spectra (black dots) at different δ_- for the two-chain model with non-Hermitian cross inter-chain coupling described by $H_{\mathrm{CNHSE-SSH}}$ in the main text. Red (green) dot lines indicate the OBC skin solution in the thermodynamic limit with a weak (zero) inter-chain coupling $\delta_{ab} = 0.5 \times 10^{-3}(0)$. Other parameters are $N = 40$, $\delta_a = -\delta_b = \delta_-$, $t_1 = 0.75$, and $V = 1.2$.

the other color as we scan k, and this new connection does not succeed in defining the expected separated two bands because the obtained eigenenergy eigenvalues jump over the seen line gap (hence indicated by "x" on the panels). This observation instructed us to take extra caution to operationally define two different bands for the intermediate case, where the two bands are not even clearly separable in the first place. Indeed, in the intermediate regime as illustrated in Fig. 6(b), the two bands are connected to form a central-loop structure, and it is subtle and ambiguous to distinguish between the two bands. Interestingly, in the case of Fig. 6(b), scanning k still yields two different schemes of connecting the eigen-energies, and we cannot exclude either connections thus obtained. We therefore operationally have two different ways of defining a band in this case. We hence compute their Berry phases γ_{r} and γ_{i} respectively corresponding to both of them. Furthermore, γ_{r} is defined following a trajectory transformed from the energy band used in Fig. 6(a), thus is expected to inherit its trivial topology. Similarly, γ_{i} possesses the same nontrivial value as that of the energy band considered in Fig. 6(c). Consistent with these understandings, our numerical calculation does yield $\gamma_{\mathrm{r}} = 0$ and $\gamma_{\mathrm{i}} = \pi$ in the intermediate regime with a point gap. For completeness, in Fig. 6(d-f), we additionally present in terms of 3-dimensional plots the above-discussed spectra versus k, obtained by solving Eq. (9).

d. Topological transition at fixed size and inter-chain couplings

Finally, we illustrate the spectra of this model with different values of δ_- in Fig. 7. By increasing δ_-, the non-reciprocity is strengthened along each chain, but toward opposite directions. Therefore, similar to the first two-chain model discussed in the main text, the effective inverse skin depth $\kappa_a - \kappa_b$ is enhanced, and we observe a transition of OBC spectrum from a line to a central-loop structure, accompanied with a topological transition reflected by the emergence of zero-energy degenerate edge states. This behavior is similar to the transition with increasing N as discussed above.

SUPPLEMENTARY NOTE 6: STABILITY OF THE CNHSE CIRCUIT AGAINST DISORDER

The circuit realization proposed in the main text involves a circuit Laplacian of the form (Eq. 5 of the main text)

$$J(k) = \begin{pmatrix} 2\omega C e^{ik} + \Delta(k) & -r^{-1} \\ -r^{-1} & 2\omega C e^{-ik} + \Delta(k) \end{pmatrix} \tag{11}$$

where $\Delta(k) = r^{-1} + 2R^{-1} - 2(R^{-1} + \omega C)\cos k$ and $\omega = 1/\sqrt{LC}$ depends on resistances r, R and capacitance C. Actual experimental setups are imperfect, but due to the robustness of non-reciprocal pumping in general, we do not expect component uncertainties to affect the spectrum significantly, as illustrated in Fig. 8 for simulated random 5% and 20% component uncertainties. To significantly modify the CNHSE spectrum, one will require extremely large component disorder of magnitudes comparable or larger than the component values themselves, which can only occur if the disorder is deliberately introduced.

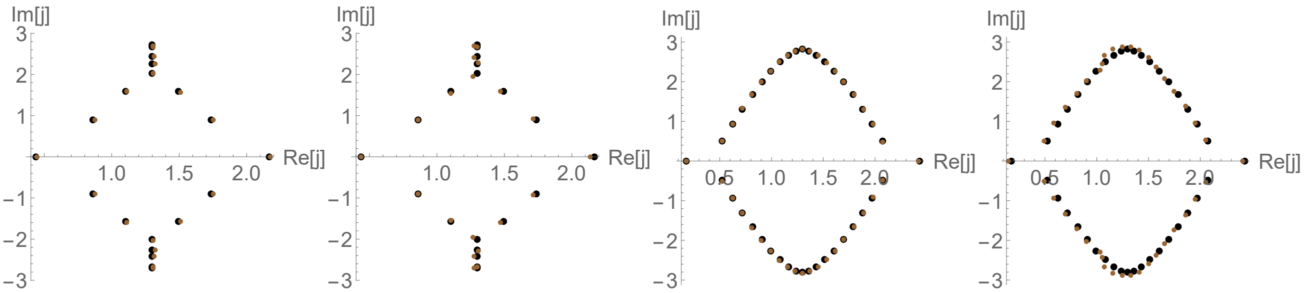

(a) (b) (c) (d)

Supplemental Figure 8. The Laplacian admittance spectra j in the clean limit (black) and with component uncertainties (brown) in all components R, L and C. (a-b) $N = 10$ unit cells, with 5% and 20% tolerances and (c-d) $N = 20$ unit cells, with 5% and 20% , all with $R = 0.6r$ and $\omega RC = 0.333$. While each system size N reveals qualitatively different spectra, as expected from the CNHSE, it is noteworthy that even a 20% component uncertainty cannot alter the spectrum to any significant degree.

SUPPLEMENTARY REFERENCES

[1] Lee, C. H. and Thomale, R., Anatomy of skin modes and topology in non-hermitian systems, Phys. Rev. B **99**, 201103 (2019).

[2] Okuma, N. and Sato, M., Topological phase transition driven by infinitesimal instability: Majorana fermions in non-Hermitian spintronics, Physical review letters **123**, 097701 (2019).

[3] Chang, P.-Y., You, J.-S., Wen, X., and Ryu, S., Entanglement spectrum and entropy in topological non-hermitian systems and non-unitary conformal field theories, Phys. Rev. Research **2**, 033069 (2020)

[4] Herviou, L., Regnault, N., and Bardarson, J. H., Entanglement spectrum and symmetries in non-Hermitian fermionic non-interacting models, SciPost Phys. **7**, 069 (2019).

[5] Mu, S., Lee, C. H., Li, L., and Gong, J., Emergent fermi surface in a many-body non-hermitian fermionic chain Phys. Rev. B **102**, 081115 (2020).

[6] Peschel, I., Calculation of reduced density matrices from correlation functions, Journal of Physics A: Mathematical and General **36**, L205 (2003).

[7] Alexandradinata, A., Hughes, T. L., and Bernevig, B. A., Trace index and spectral flow in the entanglement spectrum of topological insulators, Phys. Rev. B **84**, 195103 (2011).

[8] Lee, C. H. and Ye, P., Free-fermion entanglement spectrum through wannier interpolation, Physical Review B **91**, 085119 (2015).

[9] Yao S. and Wang, W., Edge states and topological invariants of non-Hermitian systems, Phys. Rev. Lett. **121**, 086803 (2018).

[10] Yin, C., Jiang, H., Li, L., Lü, R., and Chen, S., Geometrical meaning of winding number and its characterization of topological phases in one-dimensional chiral non-hermitian systems, Phys. Rev. A **97**, 052115 (2018).

[11] Song, F., Yao, S., and Wang, Z., Non-Hermitian skin effect and chiral damping in open quantum systems, Physical review letters **123**, 170401 (2019).

[12] Li, L., Lee, C. H., and Gong, J., Topological switch for non-Hermitian skin effect in cold-atom systems with loss, Phys. Rev. Lett. **124**, 250402 (2020).

[13] Kawabata, K., Shiozaki, K., Ueda, M., and Sato, M., Symmetry and topology in non-hermitian physics, Physical Review X **9**, 041015 (2019).

[14] Li, L., Lee, C. H., and Gong, J., Geometric characterization of non-hermitian topological systems through the singularity ring in pseudospin vector space, Physical Review B **100**, 075403 (2019).
